# Cell Division and Meristem Dynamics in Fern Gametophytes

**DOI:** 10.3390/plants12010209

**Published:** 2023-01-03

**Authors:** Xiao Wu, Xing Liu, Shaoling Zhang, Yun Zhou

**Affiliations:** 1 Department of Botany and Plant Pathology, Purdue University, West Lafayette, IN 47907, USA; 2 Purdue Center for Plant Biology, Purdue University, West Lafayette, IN 47907, USA; 3 Center of Pear Engineering Technology Research, State Key Laboratory of Crop Genetics and Germplasm Enhancement, Nanjing Agricultural University, Nanjing 210095, China; 4 Department of Biochemistry, Purdue University, West Lafayette, IN 47907, USA

**Keywords:** fern gametophyte, cell division, Ceratopteris, cellular dynamics, apical cell, multicellular meristem

## Abstract

One of the most important questions in all multicellular organisms is how to define and maintain different cell fates during continuous cell division and proliferation. Plant meristems provide a unique research system to address this fundamental question because meristems dynamically maintain themselves and sustain organogenesis through balancing cell division and cell differentiation. Different from the gametophytes of seed plants that depend on their sporophytes and lack meristems, the gametophytes of seed-free ferns develop different types of meristems (including apical cell-based meristems and multicellular apical and marginal meristems) to promote independent growth and proliferation during the sexual gametophyte phase. Recent studies combining confocal time-lapse imaging and computational image analysis reveal the cellular basis of the initiation and proliferation of different types of meristems in fern gametophytes, providing new insights into the evolution of meristems in land plants. In this review, we summarize the recent progress in understanding the cell growth dynamics in fern gametophytes and discuss both conserved and diversified mechanisms underlying meristem cell proliferation in seed-free vascular plants.

## 1. Introduction

**Figure 1:**
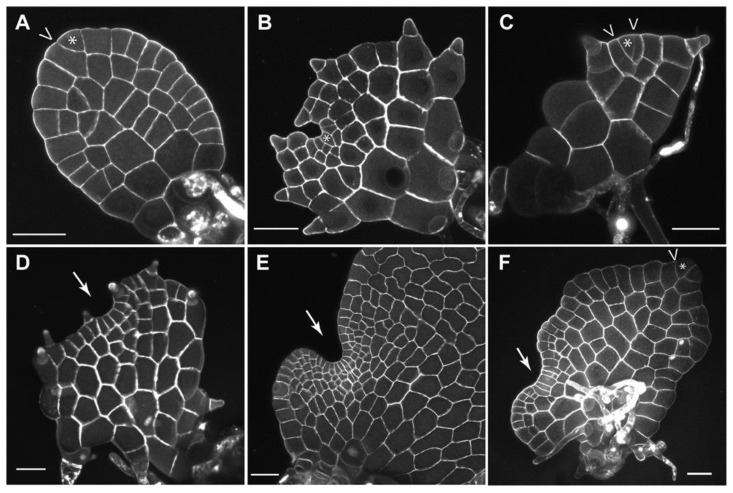
Confocal images of the three meristem types in fern gametophytes: apical cell-based (**A**–**C**,**F**), multicellular apical (**D**), and multicellular marginal (**E**,**F**). Asterisks and arrows indicate the wedge-shaped apical cells and multicellular meristems, respectively. “V” indicates the cell division in the apical cell (**A**,**C**,**F**). (**A**,**F**): *Pteris vittata*, (**B**): *Sphenomeris chinensis*, (**C**,**D**): *Woodsia obtusa*, (**E**): *Ceratopteris richardii*. Scale bar: 50 μm. Gray (**A**–**F**): cell wall stain.

**Figure 2:**
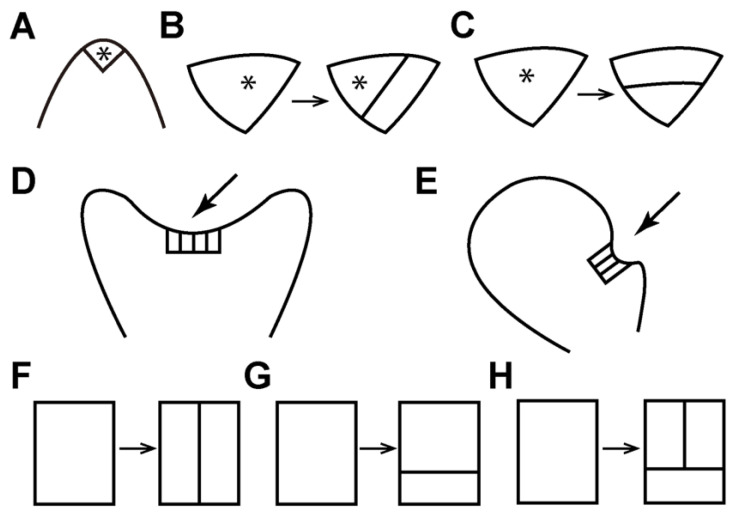
Diagrams illustrating different types of meristems and cell division patterns in fern gametophytes. (**A**–**C**) The asterisks indicate the wedge-shaped apical cells (ACs) in the AC-based meristem (**A**). The oblique division (**B**) is associated with the initiation and proliferation of ACs (**A**,**B**). The periclinal division in the ACs (**C**) usually leads to the disappearance of the ACs. (**D**,**E**) The arrows (**D**,**E**) indicate the multicellular apical meristem and the multicellular marginal meristem in fern gametophytes, respectively. (**F**–**H**) Diagrams of different patterns of cell division in multicellular meristems. The anticlinal division (**F**) is usually associated with the initiation of multicellular apical and marginal meristems. The anticlinal (**F**) and periclinal (**G**) divisions and the conserved reverse “T” type of divisions (**H**) are associated with the proliferation of multicellular apical and marginal meristems.

Composed of pluripotent stem cells, plant meristems serve as sustainable resources for organ development and body formation. In land plants, meristems have highly conserved functions in sustaining cell division, maintaining themselves as undifferentiated while continuously producing daughter cells that differentiate into different organs [1,2,3]. Thus, besides playing essential roles in plant growth and reproduction, meristems also serve as an ideal research system for studying the general principle of cell fate specification during continuous cell proliferation and organ formation. In seed plants, the sporophytes develop apical and lateral meristems, such as shoot apical meristems, root apical meristems, and vascular procambium/cambium [3,4,5,6,7]. On the other hand, their gametophytes lack any meristem and grow dependent on sporophytes [8,9]. In contrast, ferns, which are sister to seed plants [10], do not produce any seeds or flowers, and the gametophytes in ferns initiate and maintain their own meristems to drive growth independent of their sporophytes [11,12,13,14]. Compared to the well-characterized function and regulation of meristems in the sporophytes of seed plants, such as shoot apical meristems in Arabidopsis [15,16,17,18], meristem development in fern gametophytes remains underexplored [11,19]. Previous characterizations have shown that fern gametophytes develop different types of meristems, including the apical cell (AC)-based meristems and the AC-independent multicellular meristems, to sustain prothallus development and sexual organ formation [12,13,14,20,21]. Recent studies established a quantitative research platform, combining noninvasive confocal time-lapse imaging, image segmentation, and computational quantification to examine meristem development in fern gametophytes [22,23,24,25]. Using this platform, the variations in meristem development and activity were determined at single-cell resolution among different fern species, including *Ceratopteris richardii* (a model fern), *Pteris vittata* (the ladder brake), *Woodsia obtusa* (the blunt-lobe cliff fern), and *Sphenomeris chinensis* (the lace fern) [22,23,24,25]. The dynamics of different meristems (apical cell-based meristems and multicellular meristems), including their initiation, maintenance, transition between different identities, and termination during fern gametophyte development, were also quantitively examined [22,23,24,25]. In this review, we summarize the current progress in understanding the diversified meristem activities and discuss the conserved and unique division patterns that dictate meristem dynamics in fern gametophytes (Figure 1A–F and Figure 2A–H).

## 2. Apical Cells: Maintenance, Proliferation, and Disappearance

Apical cells (ACs), also called apical initials, show the iconic tetrahedral or wedge-shaped morphology and serve as the initials for cell proliferation in AC-based meristems of seed-free plant lineages (Figure 1A–C and Figure 2A) [4,14,26,27,28]. The AC-based meristems seem to be specific to seed-free plants because this unique morphology has not yet been identified in the meristems of seed plants [26]. The AC activity has been well characterized in the gametophytes of a few bryophyte species, including the moss *Physcomitrella patens* and the liverwort *Marchantia polymorpha* [29,30,31,32,33,34,35,36]. The ACs and their immediate progenies also show highly conserved morphology and functions in the sporophytes of many ferns and lycophytes, including the ferns *Nephrolepis exaltata* and *Ceratopteris richardii*, and the lycophytes *Selaginella moellendorffii* and *Selaginella kraussiana* [37,38,39,40,41]. On the contrary, in fern gametophytes, the maintenance and activity of ACs seem to be highly divergent among different taxa [12,14]. In *Ceratopteris richardii*, the species widely used as a model fern [11,19,25,42,43,44,45,46,47,48,49,50,51], the wedge-shaped AC is only transiently present at the apex of the early filamentous stage [22,25,39,52,53], which has one-dimensional growth. Such behavior was also found in gametophytes of the fern *Anemia phyllitidis*, where the AC quickly disappears after spore germination [53]. In Ceratopteris and Anemia gametophytes, the disappearance of the ACs is directly associated with the reduced and terminated division at the apices of prothalli [25,54], suggesting that the transiently maintained AC in these species does not contribute to prothallus expansion and notch formation. In contrast, recent work uncovered previously uncharacterized ACs (Figure 1B), which persistently maintain themselves during fern gametophyte development [24]. At late developmental stages, when the prothalli have established the deep apical notch and fully expanded wings, the wedge-shaped AC is still present in the gametophytes of *S. chinensis*, *Blechnum australe*, and *Cyrtomium macrophyllum*, three fern species from the order Polypodiales [24]. Computational segmentation and quantification results further demonstrated that active division and expansion of the AC and its immediate progenies contributed to notch formation and prothallus expansion in *S. chinensis* gametophytes [24]. The characterizations of ACs in different fern gametophytes altogether [14,21,22,23,24,52,54,55] suggest that the activity of wedge-shaped ACs in gametophytes likely independently evolved in fern taxa [24].

Despite the divergent activity of the ACs in different fern species, the cell division patterns that dictate or are directly associated with the renewal, maintenance, and disappearance of ACs are conserved in fern gametophytes. Independent studies all demonstrated that the conserved oblique division in the AC resulted in one new wedge-shaped apical cell and one adjacent trapezoid-shaped cell, leading to the renewal of the ACs in fern gametophytes (Figure 2B) [21,22,24]. By contrast, in the gametophytes of many fern species, including *Woodsia obtusa* and *Lygodium japonicum*, the oblique division is only maintained for a limited time [21,23]. After only a few rounds of oblique division, the AC undergoes the division in a periclinal orientation, resulting in one trapezoid-shaped daughter cell outside and one triangular daughter cell inside (Figure 2C) [23]. This type of division leads to the disappearance of the morphological signature of the ACs and likely correlates to the termination of the AC-based meristems in gametophytes (Figure 2C) [21,22,23,55].

## 3. Multicellular Apical Meristem: Transition from AC, Cell Proliferation, and Apical Notch Formation

Previous studies have demonstrated that in many fern species, a gametophyte develops the multicellular meristem that lacks any morphologically distinguishable wedge-shaped AC but includes a row of adjacent rectangular cells (Figure 1D and Figure 2D) [14]. Taking the gametophytes of *Woodsia obtusa* and *Lygodium japonicum* as examples, the two meristem identities—the AC-based meristem and the multicellular meristem—are present and functional at different developmental stages (Figure 1C,D and Figure 2A,D) [14,21,23]. The AC activity contributes to cell proliferation only at the early stages of their gametophyte development (Figure 1C), usually before establishing a deep notch [21,23]. The multicellular apical meristem, one type of multicellular meristem, initiates at the same apical region of the prothalli and directly replaces the AC-based meristem (Figure 1D and Figure 2D) [23]. After the transition, the multicellular apical meristem continues to serve as the resource for new cells at the late developmental stages and leads to prothallus expansion and notch formation [14,23]. Once established, the initial cells in the multicellular apical meristem likely maintain themselves through a cellular basis other than the AC. The asymmetric oblique division that is highly conserved in renewing the ACs is absent during the proliferation of multicellular apical meristems [21,23]. Instead, the reverse “T” type of cell division, with one periclinal division followed by an anticlinal division in the upper daughter cell, is prevalent (Figure 2H) [14,21,23]. In addition, multicellular apical meristems maintain several conserved three-celled rectangular packets, with two slender rectangular cells at the top and one short rectangular cell at the bottom [23]. The anticlinal and periclinal divisions sequentially occur in these conserved three-celled packets (Figure 2E,F), contributing to the dynamic renewal or disappearance of these packets within the multicellular apical meristem [23]. Furthermore, the quantitative study of *W. obtusa* gametophytes demonstrated that anticlinal divisions occur in both the outermost layer and the inner layer, which increases the cell number in each layer. In contrast, periclinal divisions more frequently occur in the outermost layer than in inner cells, which contributes to increasing cell layers [23].

## 4. Marginal Meristem: Active Proliferation Site Independent of ACs

In several fern species, including *Ceratopteris richardii, Pteris vittata,* and *Anemia phyllitidis*, gametophytes develop a different type of multicellular meristem that is also independent of ACs and includes a row of adjacent rectangular cells (Figure 1E,F and Figure 2E) [11,22,25,52,54]. Unlike the AC-based meristem or multicellular apical meristem located at the anterior part of gametophytes (Figure 2A,D), this type of multicellular meristem initiates with multiple anticlinal divisions in a row of adjacent cells at one lateral side of the prothalli, featuring a cluster of rectangular cells in the marginal layer (Figure 2E,F) [11,14,22,25,52,54]. Therefore, this meristem has been named the notch meristem, marginal meristem, or lateral meristem in previous studies [11,14,22,39,52,53,54], and it is referred to as the multicellular marginal meristem in this review to help distinguish it from other meristem identities.

Interestingly, though Ceratopteris and Pteris belong to the same family, the timing of the maintenance and activity for the multicellular marginal meristem and the AC in these two species is different [22]. In Ceratopteris gametophytes, the ACs quickly disappear. The multicellular marginal meristems become the center of cell division for cell proliferation and prothallus expansion, eventually leading to a typical heart-shaped structure [22,25,39,52,53]. In Ceratopteris, once the meristem notch has been established, cell division is restricted to the multicellular marginal meristem [25]. The cells outside the meristem become mitotically inactive but undergo cell expansion [25]. Within the multicellular meristems, the marginal layer has significantly higher cell division activity than the inner layer, suggesting that a positional signal activates the cell division [25]. In contrast, as shown in Figure 1F, in the majority of *P. vittata* gametophytes, both the AC-based meristem and the multicellular marginal meristem are present simultaneously but in different locations of the prothallus, even at late developmental stages [22]. Time-lapse imaging results demonstrated that in many *P. vittata* gametophytes, the AC and the multicellular marginal meristem divided within the same periods. These two meristems drive the growth in different directions, eventually resulting in highly variable prothallus morphology (Figure 1F) [22]. Similar to the multicellular apical meristem, the proliferation of marginal meristems is also driven by the reverse “T” type of cell division pattern (Figure 2H). As mentioned above, such a pattern has been identified in the gametophytes of fern species across different taxa, likely as a conserved mechanism maintaining multicellular meristem development and prothallus expansion [14,21,22,23].

## 5. Summary and Future Perspective

Time-lapse imaging and computer-assisted quantitative analysis have uncovered variations and dynamics of meristems in fern gametophytes [22,23,24,25]. These works highlighted conserved and unique cell division patterns directly associated with the initiation, maintenance, or termination of different indeterminant meristems (Figure 2) [22,23,24,25]. They also revealed the relationships among the cell position, cell size, and division activity during fern gametophyte development [23,24]. Many exciting questions derive from the current work, calling for more attention and further efforts. For instance, considering the highly diversified morphology and variable developmental processes of gametophytes in fern taxa [10,56,57,58,59,60,61], more species spanning the whole phylogeny, especially from under-represented lineages, need to be included in future studies to gain a more comprehensive view of meristem behavior and dynamics in gametophytes. In addition, future studies on key meristem regulators, such as the HAM family members [62,63,64,65,66,67] and WUS/WOX homologs [17,68,69,70], may pinpoint the potentially conserved or lineage-specific functions of these regulators and identify molecular mechanisms underlying the variations of meristem development in fern gametophytes. The roles of phytohormones and environmental signals in meristem cell proliferation in fern gametophytes also need to be explored [71,72,73,74,75]. Furthermore, computational modeling of cell division and expansion during normal and perturbed development, which integrates the in vivo time-lapse imaging results into the in silico predictions and simulation, will help understand the meristem function and evolution from a quantitative perspective. 

## Data Availability

All the data is included within the article.
